# Augmented feedback influences upper limb reaching movement times but does not explain violations of Fitts' Law

**DOI:** 10.3389/fpsyg.2015.00800

**Published:** 2015-06-16

**Authors:** John de Grosbois, Matthew Heath, Luc Tremblay

**Affiliations:** ^1^Faculty of Kinesiology and Physical Education, University of TorontoToronto, ON, Canada; ^2^School of Kinesiology, University of Western OntarioLondon, ON, Canada

**Keywords:** Fitts' Law, terminal feedback, effective target width, strategy, discrete reaching

## Abstract

Fitts' (1954) classic theorem asserts that the movement time (MT) of voluntary reaches is determined by amplitude and width requirements (i.e., index of difficulty: ID). Actions associated with equivalent IDs should elicit equivalent MTs regardless of the amplitude and/ or width requirements. However, contemporary research has reported that amplitude-based contributions to IDs yield larger increases in MTs than width-based contributions. This discrepancy may relate to the presence of augmented terminal feedback in Fitts' original research, which has not been provided in more recent investigations (e.g., Heath et al., 2011). To address this issue, participants performed reaching movements during two sessions wherein feedback regarding terminal accuracy was either provided or withheld. It was hypothesized that the absence of augmented terminal feedback would result in a stereotyped performance across target widths and explain the violation of Fitts' theorem. Yet, the results revealed distinct influences of amplitude- and width-based manipulations on MT, which also persisted across feedback conditions. This finding supports the assertion that the unitary nature of Fitts' theorem does not account for a continuous range of movement amplitudes and target widths. A secondary analysis was competed in an attempt to further investigate the violation of Fitts' Law. Based on error rates, participants were segregated into accuracy- and speed-prone groups. Additionally, target's IDs were recalculated based on each participant's performance using the effective target width (i.e., ID_We_) instead of the nominal target width. When using MT data from the accuracy-prone group with this ID_We_, the aforementioned violation was alleviated. Overall, augmented terminal feedback did not explain the violation of Fitts' theorem, although one should consider using the effective target width and participant's strategy in future investigations.

## Introduction

Fitts (1954) applied Shannon's Information Theory (Shannon and Weaver, 1949) to forward a mathematical quantification of the speed-accuracy trade-off associated with goal directed reaching actions. In particular, Fitts examined how manipulating movement amplitude (A) and target width (W) influenced the movement times (MT) of goal-directed actions. Fitts found that MT was linearly related to a target's index of difficulty (ID), which was defined as the log_2_(2A/W). The mathematical relationship between MT and ID was found to be MT = *a* + b^*^ ID, where *a* (i.e., the intercept) and *b* (i.e., the slope) are experimentally determined constants. Fitts provided seminal empirical support for the equation with both reciprocal (Fitts, 1954) and discrete goal-directed reaching movements (Fitts and Peterson, 1964). Moreover, since its inception, Fitts'^1^ theorem has been extensively examined and promulgated as a law-based measure of human motor function (e.g., Accot and Zhai, 1997; Crossman and Goodeve, 1983; Guiard and Beaudouin-Lafon, 2004).

Although Fitts' theorem has become prominent in a variety of fundamental and applied research fields, it has also faced criticism (e.g., Welford et al., 1969; Hoffmann and Sheikh, 1991; Danion et al., 1999; Adam et al., 2006; Heath et al., 2011). In what is a salient example for the present study, Heath et al. (2011) found that changes in ID induced by an amplitude-based manipulation resulted in a greater influence on MT than changes induced by a width-based manipulation. Specifically, participants performed discrete upper-limb reaches to virtual targets under a one-way mirror wherein concurrent visual feedback of the moving limb was provided via a light emitting diode (i.e., and LED) attached to the forefinger. The forefinger of participants was also tracked via a 3D optoelectric system while performing reaches to targets of varying widths (20, 30, 40, and 50 mm) and amplitudes (155, 190, 255, and 380 mm). Notably, Heath et al. (2011) employed a “width manipulation” wherein the different target widths were used across a common amplitude (i.e., 255 mm), and an “amplitude manipulation” wherein the different amplitudes were combined with a single target width (i.e., 30 mm). The resultant IDs across width and amplitude manipulations were 3.36, 3.67, 4.08, and 4.67 bits of information. The MT/ID slope for the width manipulation (13 ms/bit) was found to be significantly less than that of the amplitude manipulation (92 ms/bit), a result that is not compatible with Fitts' assertion of unitary MT/ID relations.

The purpose of the current study was to examine the influence of augmented spatial terminal feedback in replicating the tenets of Fitts' theorem. The basis for this question stems from Fitts and Peterson's (1964) work examining MT associated with discrete reaching movements to targets of varying IDs. In that work, Fitts and Peterson provided participants with explicit augmented spatial terminal feedback at the end of each trial by illuminating one of a series of three lights that indicated whether the response was accurate or exhibited an under- or over-shooting error. In contrast, Heath et al. (2011) did not provide participants with augmented spatial terminal feedback. That is, participants in the Heath et al. (2011) study received only inherent visual feedback about the spatial position of their limb (i.e., represented by the LED) compared to the virtual target. This is a potentially important distinction between the aforementioned studies because terminal feedback often plays an important role in everyday movements and could influence target width utilization. Indeed, goal-directed reaching movements in everyday life often lead to noticeable effects on the environment. For example, when individuals reach out to press a button on a keyboard, they receive immediate visual feedback on the computer screen regarding their movement accuracy. In contrast, contemporary examinations of Fitts' equation often rely on virtual displays that are relatively static and unchanging (e.g., Guiard and Beaudouin-Lafon, 2004; Zhai et al., 2004; Heath et al., 2011; Blinch et al., 2012). Thus, even if an experimenter can accurately localize a participant's finger with a motion tracking system, the completion of such an action in a virtual environment provides less information as to whether or not their finger has made it adequately onto the target. That is, in the absence of augmented feedback about movement outcome, participants may adopt strategies unlike those utilized in day to day life. As such, Schmidt et al. (1985) found that participants reduced their movement endpoint variability at the expense of MT, when provided with spatially determined feedback. Thus, the prioritization of speed or accuracy could theoretically be influenced by the presence or absence of spatial terminal feedback. Consequently, the lack of augmented spatial terminal feedback may alter endpoint accuracy and influence speed/accuracy trade-offs. For instance, in the absence of spatial terminal feedback, participants may adopt a conservative strategy to utilize only a small proportion of the target area in order to confidently complete the task successfully.

Another potentially important aspect of speed/accuracy trade-offs pertains to the strategy employed by the participant during action. Adam (1992) reported the presence of individual differences in the prioritization of speed and accuracy. That is, when completing reciprocal reaching movements, some individuals adopted strategies to prioritize speed while others prioritized accuracy. Also, Adam (1992) found that this prioritization of speed or accuracy could be influenced by providing instructions. Other studies have also shown how instructions can influence movement planning and control mechanisms (e.g., Elliott et al., 1991; Rival et al., 2003; Lin et al., 2008; Young et al., 2009; Massie and Malcolm, 2012). Yet, it remains that the increased use of virtual environments to test Fitts' theorem could have influenced how participants prioritize or strategize on their movement speed vs. accuracy.

Recall that Heath et al. (2011) employed a virtual reaching environment which entails two methodological nuances: (1) participants were provided with an impoverished representation of their reaching limb (i.e., it was rendered via a LED); and (2) participants were not able to physically touch the target object (i.e., because it was a virtual rendering). When the movement endpoint was close to the target boundary, participants may not have been certain that the finger adequately landed onto the target area. Thus, the different target widths may have yielded relatively small differences in endpoint variability. This could result in an under-utilization of the target area across trials. In line with this prediction, Heath et al. reported that the two-dimensional endpoint variability for visually guided movements toward the 50 mm target width was 472 mm^2^, which represents 24% of the 1963 mm^2^ target area (see Heath et al., 2011; Table 1). In contrast, the endpoint variability for the 20 mm target width was of 314 mm^2^, which represents 100% of the 314 mm^2^ target area (see Heath et al., 2011; Table 1). To take into account these performance differences across target widths, the effective target width (W_e_: i.e., 95% confidence interval of the endpoints) was calculated to obtain the associated effective index of difficulty (i.e., ID_We_). ID_We_ ranged from 5.18 to 5.31 bits for the width manipulations, and 4.66–5.80 bits for the amplitude manipulations. The lack of ID_We_ scaling for the width manipulation may suggest that the reduced MT/ID scaling to width-based manipulations reflects a general under-utilization of target widths in discrete reaching movements. This explanation is in line with the observed average MT ranging from 358 to 376 ms for the width manipulations and from 295 to 415 ms for the amplitude manipulations (see Heath et al., 2011; Table 1). As such, the steeper MT/ID slopes for amplitude than width manipulations (Heath et al., 2011) may be due to a more stereotyped endpoint variability caused by the lack of knowledge regarding endpoint accuracy.

In the present study, we examined if the discrepant MT/ID slopes associated with amplitude- and width-based manipulations used by Heath et al. (2011) were related to the absence of augmented spatial terminal feedback. To accomplish that objective, participants completed discrete reaching movements in separate blocks that entailed the presentation of augmented spatial terminal feedback (as in Fitts and Peterson, 1964) and a separate block wherein such feedback was not provided (as in Heath et al., 2011). In terms of research predictions, it was hypothesized that if the introduction of augmented terminal feedback produces a larger difference in endpoint variability for the width manipulation, then larger changes in MTs should also be observed. Therefore, MT/ID slopes for amplitude manipulations are predicted to be steeper than MT/ID slopes for width manipulations, but only in the absence of augmented terminal feedback. In contrast, if augmented terminal feedback does not modulate speed/accuracy relations then amplitude-based manipulations should yield steeper MT/ID slopes than width-based manipulations regardless of the availability of augmented spatial terminal feedback.

## Methods

### Participants

Nineteen participants with self-reported normal or corrected-to-normal vision were recruited (7 male, 12 female; age range: 18–33). Sixteen participants were right-handed and three were left-handed. All participants gave informed written consent prior to the experiment and were compensated $14 CAD. The research project was approved by, and performed in accordance to the ethical standards of the University of Toronto Office of Research Ethics.

### Apparatus and procedures

**Figure 1:**
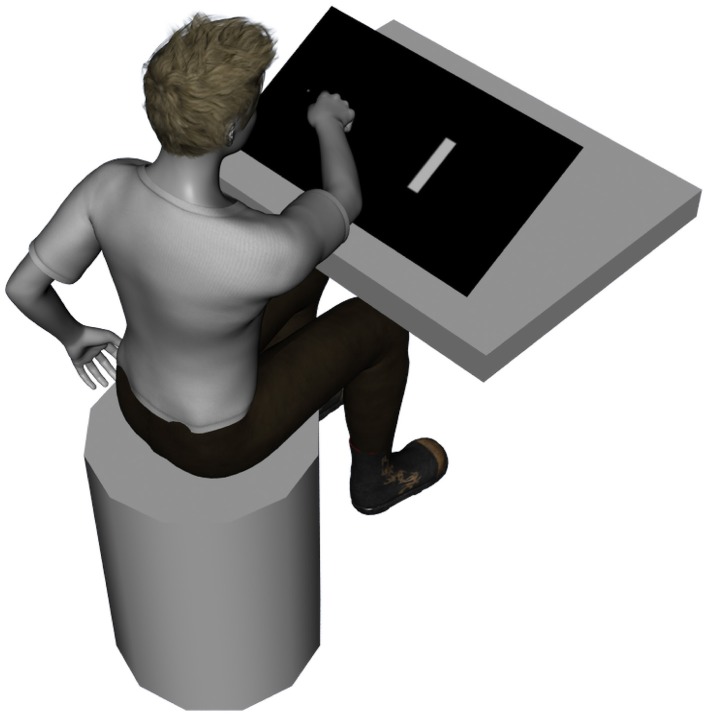
**Depiction of the experimental setup**.

The experiment was conducted in a dimly lit (4 lux or cd/m^2^) and quiet room (38 dB). Participants were seated comfortably at a table, upon which a 22 inch wide-screen LCD monitor was positioned (Model: GD23HZ; Resolution: 1280 × 1024 pixels; @ 60 Hz; ACER Inc.). The far edge of the screen was raised off the tabletop resulting in the screen surface being pitched at approximately 30° from the horizontal plane (i.e., table surface), toward the participant (see Figure 1 for a schematic representation). The center of the screen was aligned with the mid-sagittal plane of the participant. A sheet of transparent polymer was placed upon the monitor surface to allow for the display surface to be touched without distorting images.

**Figure 2:**
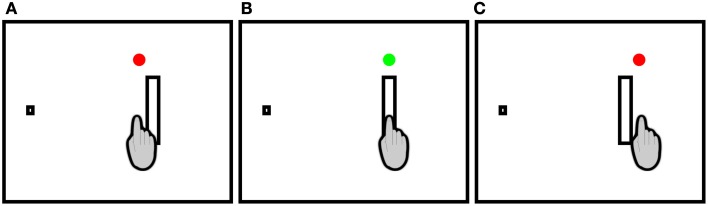
**Feedback possibiliies in the TF-Present condition**. Participants made rightward discrete pointing movements to rectangular targets. A red circle appeared immediately following the movement to indicate **(A)** undershoots and **(C)** overshoots while a green circle appeared above the target to indicate **(B)** accurate movements. In the TF-Absent condition, no circles appeared following the movements.

Each participant completed two sessions of reaching trials separated by at least 24 h. One of the sessions was completed with augmented spatial terminal feedback (TF) after each movement (TF-Present), using a form of feedback analogous to Fitts and Peterson (1964), and the other session was completed in the absence of augmented spatial terminal feedback (TF-Absent). The ordering of sessions was randomized across participants. In the TF-Present session, upon movement completion feedback was given in one of three forms: (1) When the movement was accurate, a green circle appeared above the target (see Figure 2B); (2) When an undershooting error occurred a red circle appeared above and closer to the home position (see Figure 2A); and (3) When an overshooting error a red circle appeared above and further away from the home position (see Figure 2C).

Each trial began with the presentation of a home position on the left side of the screen (i.e., a 10 by 10 mm filled white square against a black background). Participants placed their right index fingertip upon the home position after which time the experimenter initiated the trial sequence. Following a 1–2 s variable foreperiod, a target was presented to the right of the home position. The targets were filled white bars against a black background. Four levels of target amplitude (155, 190, 266, and 380 mm) and target width (20, 30, 40, and 50 mm) were utilized. The target bars were all 101 mm in height (i.e., dimension orthogonal to the width). Each of the four target amplitudes were orthogonally combined with each of the four target widths to yield 16 conditions and a range of IDs from 2.63 to 5.25 bits (see Table 1). The appearance of the target signaled to the participant to initiate their reaching movement “as quickly and accurately as possible.” During the TF-Present session, augmented spatial terminal feedback was displayed on the screen immediately following the movement end (see Figures 2A–C for a schematic representation; see below for a description of movement end criteria). The augmented spatial terminal feedback and target was available upon movement completion until the end of the 1.5 s collection period plus an additional 0.5 s. During the TF-Absent session, participants completed their movements to the targets and were not provided with the additional augmented spatial terminal feedback. Regardless of session, the end of the trial was signaled by the removal of the target from the screen, which informed the participants to return to the home position for the next trial. Stimulus presentation was controlled using a custom Matlab script (The MathWorks Inc.) and PsychToolBox 3 (Brainard, 1997).

**Table 1:** **Indices of difficulty of the employed 16 targets (in bits) as well as the average index of difficulty across amplitude and width manipulations**.

**Widths (mm)**	**Amplitudes (mm)**				**Average ID (bits)**
	**155**	**190**	**266**	**380**	
**20**	3.95	4.25	4.73	5.25	4.55
**30**	3.37	3.66	4.15	4.66	3.96
**40**	2.95	3.25	3.73	4.25	3.55
**50**	2.63	2.93	3.41	3.93	3.23
**Average ID (bits)**	3.23	3.52	4.01	4.52	

Ten practice trials were performed at the beginning of each session and served as a familiarization period. Following the practice trials, participants completed 10 trials to each of the 16 amplitude/width combinations (i.e., 4 amplitudes by 4 widths), arranged in 16 blocks of 10 trials (i.e., 160 experimental trials per session). The order of the blocks was pseudo-randomized, with the limitation that the IDs of the amplitude/width combinations of the first eight blocks were within 1 bit of the IDs of the combinations employed in the last eight blocks. This limitation was to ensure that any potential effects of fatigue would be distributed comparably across IDs.

An infrared light emitting diode (IRED) was affixed to the dorsal surface of the tip of the participants right index finger and position data from the IRED were measured via an Optotrak Certus infrared camera system (Northern Digital Inc., Waterloo, ON). The three-dimensional position of the IRED was recorded at 200 Hz. The displacement data was gathered with a custom Matlab script.

### Data reduction

Instantaneous velocity profiles were calculated from the displacement data using first order differentiation. Movement start was determined as the first sample after which the finger velocity was above 30 mm/s for five consecutive samples, and movement end was determined as the first sample after which the finger velocity fell below 30 mm/s for five consecutive samples. Any movements initiated after the first triggering of the movement end criteria (e.g., delayed corrections or early return toward the home position) were not considered for further analysis because the feedback had already been provided. This is the case because any terminal feedback did appear on the screen within approximately 37 ms after the finger velocity fell below 30 mm/s.

All dependent measures were calculated along the primary (i.e., horizontal) movement axis. These variables included movement time (MT: the time between movement start and end), as well as constant error (CE: average endpoint bias), and variable error (VE: standard deviation of endpoints).

MT/ID slopes were quantified separately for amplitude and width manipulations. For the amplitude-based manipulation, four MT/ID slopes (i.e., *b*_amplitude_) were computed as a function of target width (i.e., one slope for each width). Likewise, for the width-based manipulation, four MT/ID slopes (i.e., *b*_width_) were computed separately as a function of amplitude (i.e., one for each amplitude). These slopes (*b*_amplitude_, *b*_width_) are henceforth referred to as Component Slopes.

The Component Slopes were also calculated in two different fashions. One set of slopes was computed using Fitts' ID [i.e., ID = log_2_(2A/W)], and thus yielded MT/ID slopes (i.e., *b*_Fitts_). Another set was computed using effective target width W_e_ [ID_We_ = log_2_(2A/W_e_)], and thus yielded MT/ID_We_ slopes (i.e., *b*_We_). The W_e_ was calculated using the 95% confidence interval for endpoints in the primary movement axis (i.e., 4.13 ^*^ the standard deviation of endpoints; see Welford, 1960). The use of effective target width could theoretically circumvent the potential influence of target under-utilization. That is, participants may not make use of the entire target width. Thus, the computation of ID_We_ slopes was deemed relevant to the current study because Heath et al. (2011) observed an under-utilization of target area which resulted in a much smaller range of ID_We_ (5.18–5.31 bits) for width manipulations (cf. 4.66–5.80 bits for amplitude manipulations) than ID (3.36–4.67 bits).

### Statistical analysis

MT, CE, and VE data were submitted to 2 TF-Condition (TF-Absent and TF-Present) by 4 Amplitude (155, 190, 266, and 380 mm) by 4 Width (20, 30, 40, and 50 mm) mixed-model ANOVAs. Data computed for each set of slopes (i.e., *b*_Fitts_ and *b*_We_) were analyzed separately via 2 Component Slope (*b*_amplitude_ and *b*_width_) by 4 Average ID (see Table 1)^2^ repeated measures ANOVAs. Statistical analysis was completed using R (R Core Team, 2014), and the ANOVAs were computed with the EZ package (Lawrence, 2013). *P*-values were reported to three decimal places, and effect sizes were reported as the generalized eta-squared (η^2^_G_: see Olejnik and Algina, 2003; Bakeman, 2005). When the assumption of sphericity was violated, the Greenhouse-Geisser correction was applied and the corrected degrees of freedom were reported to the nearest decimal. *Post-hoc* procedures for significant main effects and interactions involving the continuous variables of Amplitude or Width for the MT, CE, and VE analyses were submitted to polynomial contrasts using orthogonal weights accounting for the unequal spacing between amplitudes or widths (see Grandage, 1958; Carmer and Seif, 1963). Alpha was set at 0.05 for all analyses.

## Results

### Movement time

**Figure 3:**
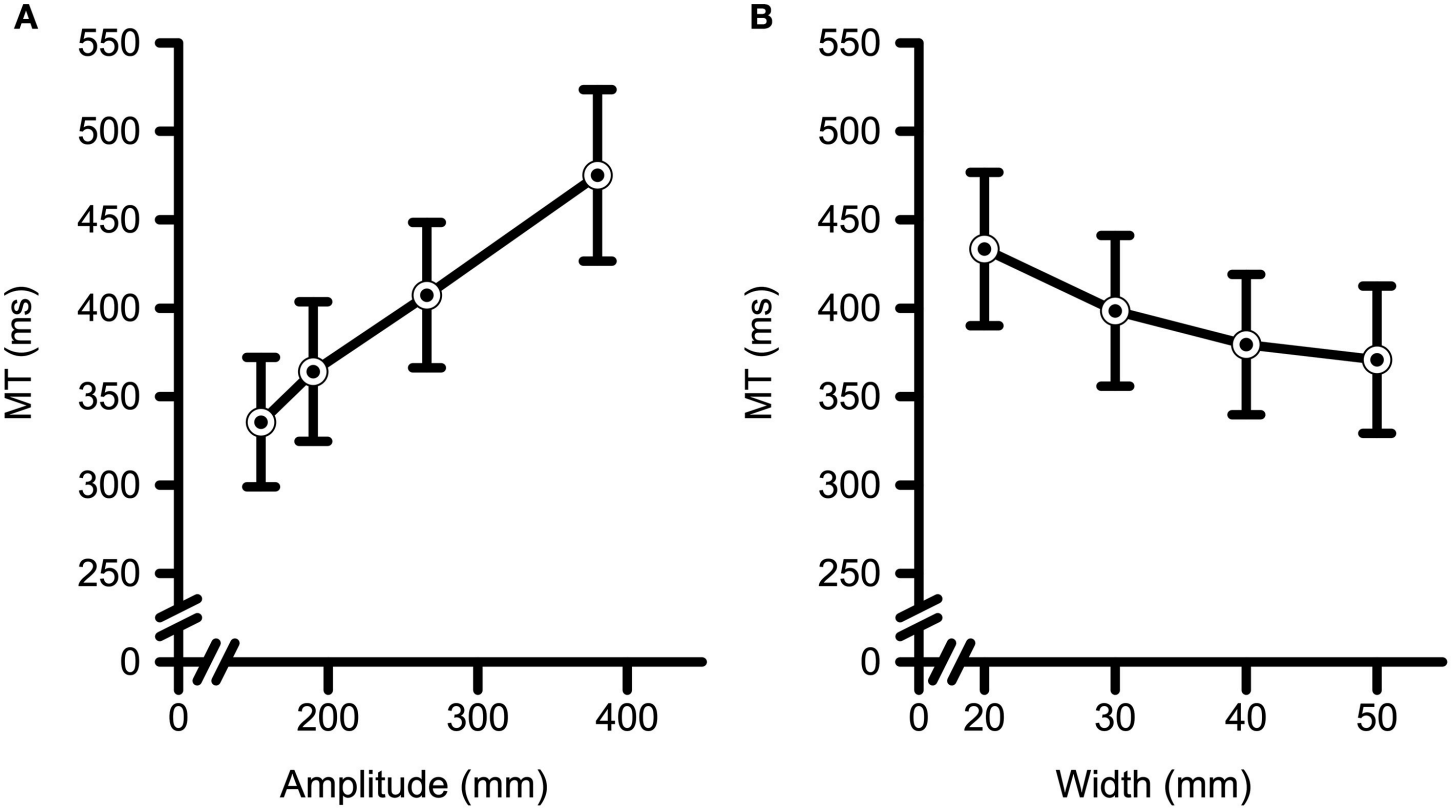
**Movement time (i.e., MT) data as a function of target (A) amplitude and (B) width**. MT increased with increases in target amplitude and decreases in target width. Error bars represent ±2 SEM.

A summary of all MT data can be found in Table 2. Main effects were found for TF-Condition [*F*_(1, 18)_ = 5.27, *p* = 0.034, η^2^_G_ = 0.01], Amplitude [*F*_(1.4, 24.3)_ = 218.60, *p* < 0.001, η^2^_G_ = 0.23], and Width [*F*_(1.3, 23.2)_ = 34.53, *p* < 0.001, generalized η^2^_G_ = 0.06]. The main effect of TF-Condition revealed that average MT was shorter in the TF-Present condition (*M* = 384 ms, *SD* = 88) than in the TF-Absent condition (*M* = 407 ms, *SD* = 96). *Post-hoc* polynomial contrasts of the main effect of Amplitude indicated that MT increased with a significant linear trend with increases in Amplitude [*F*_(1, 18)_ = 259.10, *p* < 0.001, η^2^_G_ = 0.26; see Figure 3A]. *Post-hoc* polynomial contrasts of the main effect of Width indicated that significant linear [*F*_(1, 36)_ = 69.72, *p* < 0.001, η^2^_G_ = 0.06] and quadratic [*F*_(1, 36)_ = 5.57, *p* = 0.024, η^2^_G_ = 0.01] trends were present. This could be seen in Figure 3B through the decreasing yet leveling off of MT with increasing Width.

**Table 2:** **Mean movement time in ms (and SD) across TF-Conditions, participants' strategy group, target widths and movement amplitudes**.

**Amplitudes (mm)**	**Accuracy-prone**				**Speed-prone**			
	**Widths (mm)**				**Widths (mm)**			
	**20**	**30**	**40**	**50**	**20**	**30**	**40**	**50**
**TF-ABSENT**								
**155**	434 (73)	396 (68)	360 (101)	363 (91)	290 (51)	289 (48)	268 (54)	269 (66)
**190**	459 (78)	421 (99)	389 (85)	394 (102)	308 (51)	305 (48)	306 (55)	294 (50)
**266**	528 (103)	469 (95)	430 (83)	442 (103)	356 (48)	338 (53)	346 (45)	329 (52)
**380**	602 (106)	554 (138)	514 (106)	495 (117)	426 (50)	384 (69)	374 (60)	370 (59)
**TF-PRESENT**								
**155**	399 (88)	359 (85)	327 (79)	321 (94)	292 (38)	277 (38)	270 (56)	260 (51)
**190**	431 (96)	394 (97)	368 (98)	347 (96)	310 (58)	299 (44)	303 (44)	292 (48)
**266**	469 (70)	425 (91)	420 (105)	404 (90)	341 (34)	329 (65)	339 (53)	325 (49)
**380**	562 (104)	508 (98)	487 (126)	471 (108)	432 (20)	391 (45)	387 (45)	364 (53)

### Constant and variable error

**Figure 4:**
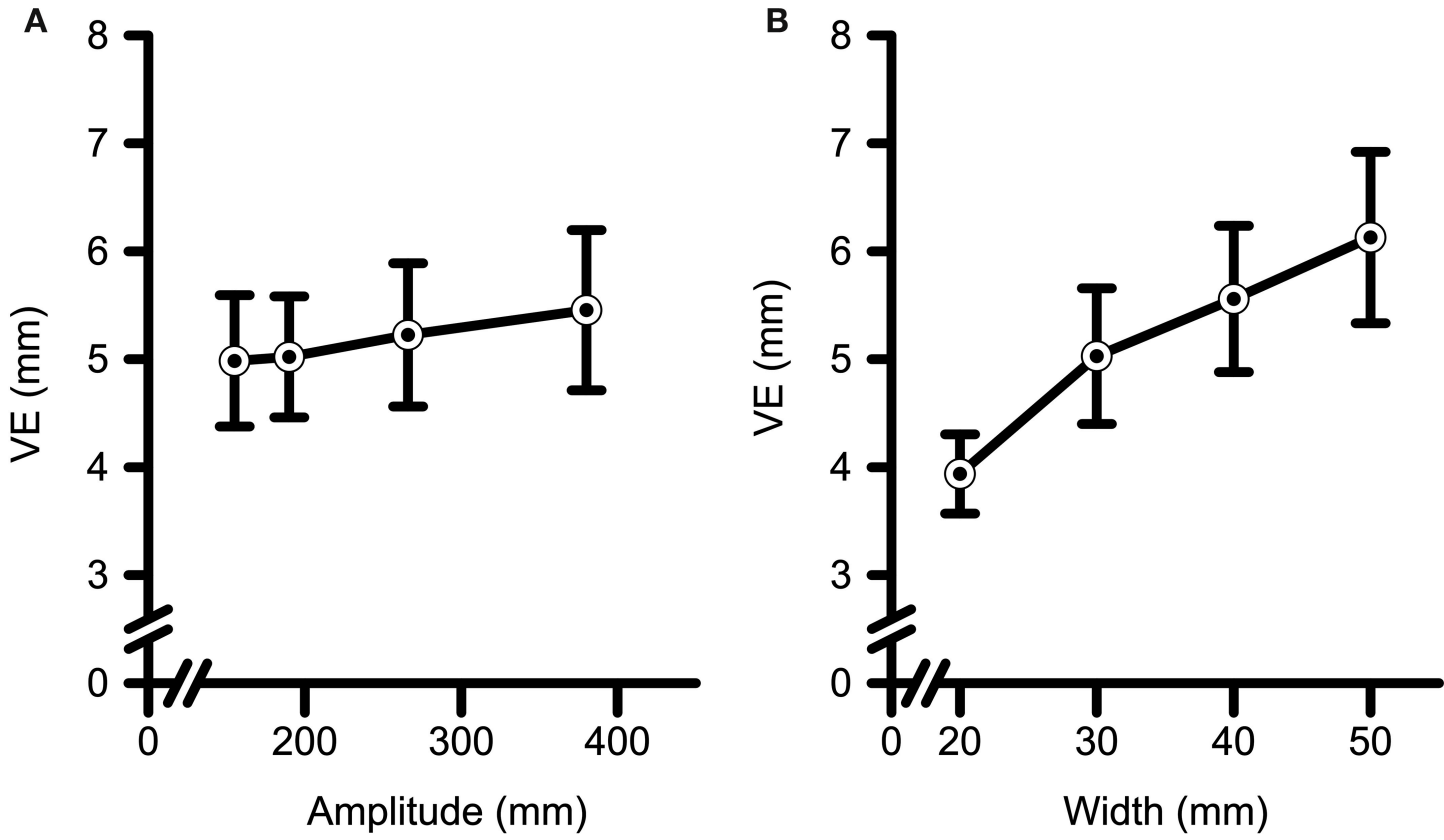
**Variable error (i.e., VE) data as a function of target (A) amplitude and (B) width**. VE increased with increases in target amplitude, and with increases in target width. Error bars represent ±2 SEM.

The CE analyses (see Table 3 for a summary of all data) a main effect of Amplitude [*F*_(3, 54)_ = 3.43, *p* < 0.023, η^2^_G_ = 0.01] and an interaction between Amplitude and Target Width [*F*_(9, 162)_ = 2.20, *p* = 0.025, η^2^_G_ = 0.01]. *Post-hoc* polynomial contrasts were completed across Amplitudes within each level of Width. Significant linear [*F*_(1, 36)_ = 5.94, *p* = 0.020, η^2^_G_ = 0.03], and quadratic [*F*_(1, 36)_ = 4.22, *p* = 0.047, η^2^_G_ = 0.02] trends were observed across increasing Amplitudes only for the 50 mm Width. Participants tended to overshoot the closest two Amplitudes and undershoot the farther two Amplitudes with a maximal undershooting observed at the 266 mm Amplitude. The analyses of VE (see Table 4 for a summary of all data) yielded significant main effects for Amplitude [*F*_(3, 54)_ = 2.84, *p* = 0.046, η^2^_G_ = 0.01], and Width [*F*_(1.6, 29.3)_ = 41.52, *p* < 0.001, η^2^_G_ = 0.16]. Polynomial contrasts of the main effect of Amplitude (See Figure 4A) determined that VE increased with a linear trend with increasing Amplitude [*F*_(1, 18)_ = 7.81, *p* < 0.012, η^2^_G_ = 0.02]. Polynomial contrasts of the main effect of Target Width (See Figure 4B) determined that VE increased with a significant linear trend with increasing in Target Width [*F*_(1, 18)_ = 53.84, *p* < 0.001, η^2^_G_ = 0.26].

**Table 3:** **Constant error in mm (and SD) across TF-Condition, participants' strategy group, target widths and target amplitudes**.

**Amplitudes (mm)**	**Accuracy-prone**				**Speed-prone**			
	**Widths (mm)**				**Widths (mm)**			
	**20**	**30**	**40**	**50**	**20**	**30**	**40**	**50**
**TF-ABSENT**								
**155**	0.29 (2.16)	1.11 (1.93)	0.48 (3.36)	1.07 (2.47)	1.19 (4.30)	0.88 (3.74)	0.89 (2.77)	0.29 (2.16)
**190**	0.09 (2.06)	−0.36 (2.64)	0.67 (2.72)	0.49 (1.83)	−0.11 (2.70)	0.75 (2.92)	2.04 (4.38)	0.09 (2.06)
**266**	0.79 (1.88)	0.50 (2.74)	0.57 (2.57)	−1.41 (3.70)	1.09 (2.30)	0.86 (2.45)	−0.01 (3.16)	0.79 (1.88)
**380**	0.30 (1.66)	−0.01 (2.48)	−0.42 (2.41)	−1.20 (3.72)	1.37 (1.68)	1.64 (3.60)	0.31 (3.90)	0.30 (1.66)
**TF-PRESENT**								
**155**	0.42 (1.44)	0.03 (1.45)	0.15 (2.69)	−0.93 (2.33)	0.46 (1.23)	1.41 (2.27)	0.11 (3.65)	0.42 (1.44)
**190**	−0.07 (1.37)	−0.23 (2.27)	0.33 (2.18)	−0.07 (3.21)	0.25 (1.20)	0.10 (2.86)	0.20 (3.96)	−0.07 (1.37)
**266**	−0.05 (1.91)	−0.98 (2.55)	−0.22 (2.04)	−1.56 (3.72)	0.56 (1.25)	−0.52 (2.59)	−0.87 (4.07)	−0.05 (1.91)
**380**	0.27 (2.51)	0.09 (2.43)	1.07 (2.83)	−0.27 (2.99)	−0.34 (1.92)	−1.32 (2.45)	0.85 (2.80)	0.27 (2.51)

**Table 4:** **Variable error in mm (and SD) across TF-Condition, participants' strategy group, target widths and target amplitudes**.

**Amplitudes (mm)**	**Accuracy-prone**				**Speed-prone**			
	**Widths (mm)**				**Widths (mm)**			
	**20**	**30**	**40**	**50**	**20**	**30**	**40**	**50**
**TF-ABSENT**								
**155**	3.45 (0.94)	4.21 (1.47)	4.58 (1.75)	4.65 (1.69)	3.55 (1.20)	5.95 (1.95)	5.34 (1.74)	6.65 (2.77)
**190**	3.52 (0.65)	3.86 (1.60)	4.67 (1.84)	4.85 (1.27)	4.92 (1.46)	4.85 (1.78)	7.04 (1.91)	6.53 (2.04)
**266**	3.09 (1.09)	4.19 (1.94)	5.07 (1.50)	5.41 (1.37)	4.89 (1.52)	5.39 (1.60)	6.40 (1.94)	8.40 (3.15)
**380**	3.47 (1.14)	4.11 (1.75)	5.58 (1.81)	6.13 (2.56)	5.17 (0.73)	6.61 (1.58)	7.14 (2.33)	7.79 (4.32)
**TF-PRESENT**								
**155**	3.61 (1.26)	5.45 (1.41)	4.99 (2.11)	6.46 (2.89)	4.15 (1.26)	5.59 (1.92)	6.45 (2.11)	6.46 (2.66)
**190**	3.99 (1.13)	4.68 (1.55)	5.65 (2.39)	5.72 (2.22)	4.38 (1.42)	5.64 (1.62)	6.20 (1.18)	6.16 (1.97)
**266**	3.59 (1.07)	4.41 (1.30)	5.49 (2.63)	5.66 (2.50)	5.28 (1.01)	7.25 (1.89)	5.61 (2.20)	7.00 (1.66)
**380**	3.31 (1.11)	5.03 (1.51)	4.78 (1.76)	6.04 (2.38)	4.96 (0.75)	6.34 (1.68)	6.76 (1.93)	7.79 (2.62)

### Slopes analyses: *b*_Fitts_ and *b*_We_

**Figure 5:**
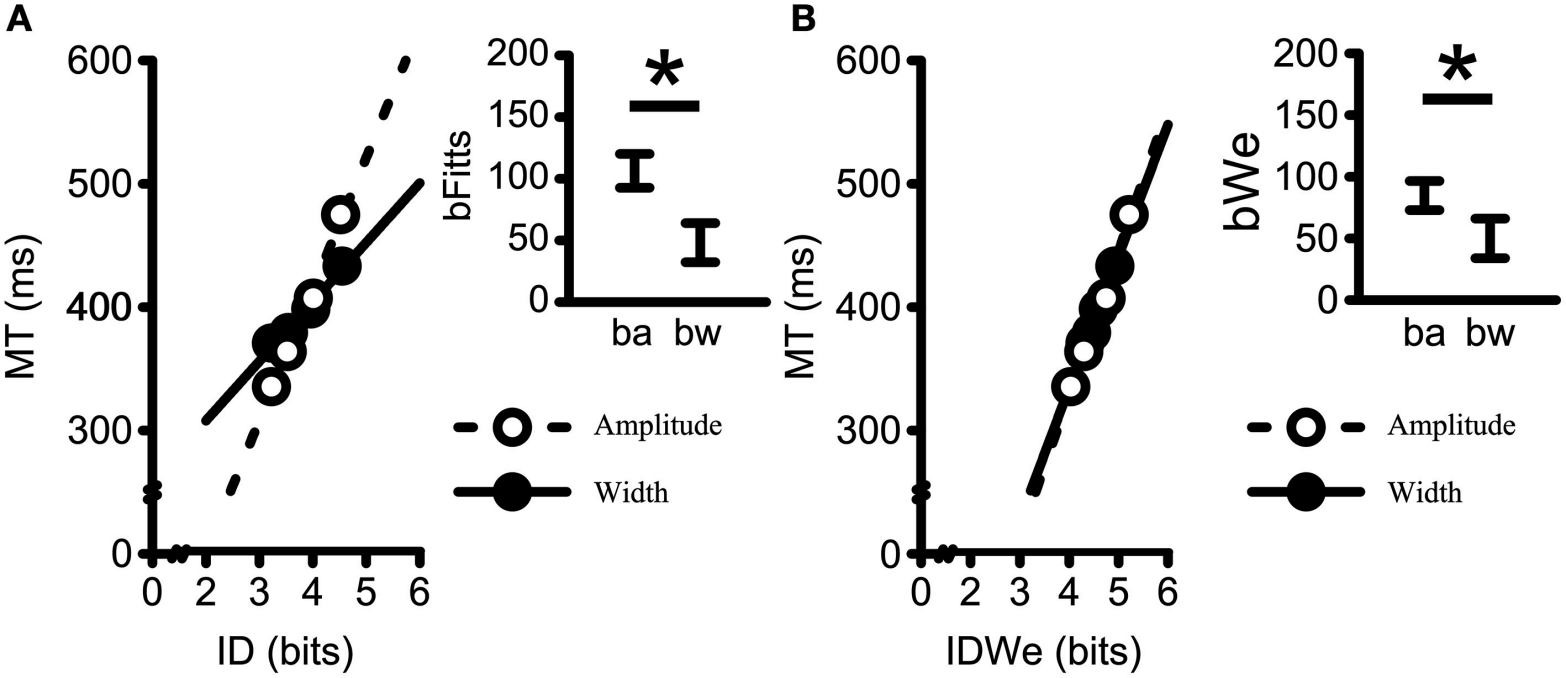
**Mean movement times plotted against (A) average ID (i.e.,** ***b*****_Fitts_); and (B) ID_We_, (i.e.,** ***b*****_We_) for all participants collapsing across terminal feedback, amplitudes within widths, and widths within amplitudes**. Dashed lines represent linear regressions on the Amplitude data; Solid-lines represent linear regression on the Width data (All *R*^2^-values > 0.99). Insets represent the 95% confidence interval of the average within participant slopes (*b*_a_ = *b*_amplitude_; *b*_w_ = *b*_width_). The movement time scaling to changes in amplitude resulted in steeper slopes as compared to the movement time scaling to changes in width for both the standard formulation of Fitts' Law (i.e., *b*_Fitts_) and Fitts' Law when using the the effective target width formulation (i.e., *b*_We_). ^*^Denotes a significant difference at *p* < 0.05.

Figures 5A,B presents the influence of amplitude- and width-based manipulations on MT/ID relations. The main graphs represent the Mean MT vs. ID (Figure 5A) and the Mean MT vs mean ID_We_ (Figure 5B) as a function of target amplitude and target width. The insets represent the 95% confidence intervals of the respective slopes as a function of target amplitude (i.e., *b*_a_) and target width (i.e., *b*_w_). The analysis of the *b*_Fitts_ slopes (i.e., MT/ID) yielded a significant main effect of Component Slope, [*F*_(1, 18)_ = 55.19, *p* < 0.001, η^2^_G_ = 0.30] (see Figure 5A). The *b*_Fitts_ relationship was steeper for the amplitude-based (*M* = 106 ms/bit, *SD* = 28) as compared to the width-based manipulation (*M* = 48 ms/bit, *SD* = 33). Likewise, the analysis of the *b*_We_ slopes (i.e., MT/ID_We_) also yielded a significant main effect of Component Slope [*F*_(1, 18)_ = 22.74, *p* < 0.001, η^2^_G_ = 0.07] (see Figure 5B) with steeper *b*_We_relations for the amplitude-based manipulation (*M* = 85 ms/bit, *SD* = 24) as compared to the width-based manipulation (*M* = 50 ms/bit, *SD* = 33). Notably, however, both *b*_Fitts_ and *b*_We_ component slopes were not significantly influenced by the provision of augmented terminal spatial feedback (*F*s < 0.52, *p*s > 0.670).

## Discussion

The primary aim of the current study was to test whether the provision of visual augmented spatial terminal feedback elicits unitary MT/ID relationships across manipulations of width and amplitude. As hypothesized, the provision of augmented spatial terminal feedback yielded shorter MTs. Most notably, however, MT/ID slopes for amplitude-based manipulations were steeper than for the width-based manipulations regardless of the availability of augmented spatial terminal feedback.

### Augmented spatial terminal feedback

The shorter average MT observed in the presence of augmented spatial terminal feedback was hypothesized and has been previously reported (i.e., Hatfield et al., 2010). Indeed, Hatfield et al. (2010) observed that more recent incarnations of Fitts' reaching paradigm (e.g., displacing a cursor on a screen) do not provide much feedback about endpoint accuracy. To test the importance of spatial terminal feedback, they asked participants to complete reciprocal reaching movements with a cursor to 3 pairs of targets of differing IDs (4.03, 5.07, and 7.64 bits). The amplitude of the movements was held constant across IDs and only the target width was manipulated. Each time the cursor entered a target's area, auditory feedback could be given in the form of a 500 ms tone. The presence of auditory feedback yielded shorter average MT and shorter time spent in the deceleration phase of the movement. Hatfield et al. (2010) posited that providing an auditory cue once the cursor entered the target's area facilitated earlier and more efficient movement reversals. Notably, however, Hatfield et al.'s explanation does not provide a framework for accounting for the present results because the discrete reaching responses of this study did not entail a movement reversal.

Another possible account relates to how individuals encode errors. In particular, on trials without augmented feedback, it is possible that the participants in the current study encoded some accurate endpoints as “target misses.” For example, if a participant's implicit goal was to place both edges of the finger into the target area, then some trials could have been perceived as an error even when the centroid of IRED (i.e., which provides the computer-based measure of accuracy) was within the target area. As such, the augmented spatial terminal feedback may have facilitated the participant's own error labeling process and resulted in a more precise target representation (e.g., Schmidt, 1975). Such a fast movement strategy could emerge because the participants could be more confident they would hit the target when terminal feedback was provided. Perhaps future work employing erroneous terminal feedback (e.g., Buekers et al., 1992) would help confirm this proposal. Overall, augmented feedback about movement endpoints may enhance the representation of the target when reaching in virtual environments.

### Amplitude vs. width slopes

The two main findings regarding the amplitude- and width-based slopes analyses were: (1) no effect of terminal feedback on width- and amplitude-based slopes; and (2) steeper slopes for amplitude- than for width-based manipulations (i.e., *b*_amplitude_ > *b*_width_) when employing either Fitts' classic representation of ID (*b*_Fitts_) or an ID based on effective target width (*b*_We_) (see Heath et al., 2011). The performance of individuals was more consistent with Welford et al. (1969), who proposed a formula that employs two separate slopes when calculating speed-accuracy trade-offs: One slope based on amplitude changes and one slope based on width changes [MT = *a* log_2_(A) + *b* log_2_(1/W), where *a* and *b* are slopes; see also Medina et al., 2009]. The steeper slope for amplitude- as compared to width-based manipulations in the current study clearly show that MT/ID relations are not unitary, and our results support Welford's proposal that the accurate prediction of MT requires a theorem that separately accounts for changes in MT as a function of an amplitude- and width-based manipulations.

Overall, despite the above-mentioned changes in MT as a function of terminal feedback, the MT/ID slopes did not reliably differ across terminal feedback conditions. Interestingly, however, when considering the plotted mean relationships (i.e., collapsing across TF-Conditions, amplitudes within widths, and widths within amplitudes) in Figure 5 (i.e., panel B main plot), the amplitude- and width-based slopes for *b*_We_ formulation do not appear to be visibly different. Nevertheless the statistical analysis on the *b*_We_ data yielded significant differences between *b*_amplitude_ and *b*_width_ slopes. The graphical similarity in the slope of the means is due to the fact that when both the ordinate and abscissa vary for each data point, as is the case in Figure 5B, the slope of the means (i.e., of each participant's data points; see Figure 5B main plot) does not equal the mean of the slopes of each participant (i.e., see Results Section and Figure 5B inset). This discrepancy led to further considerations as to the cause of the violation of Fitts' theorem. Because of this convergence of *b*_amplitude_ and *b*_width_ slopes for *b*_We_ (i.e., Figure 5B main plot), special consideration was given to *b*_We_. In addition, because each participants' strategy (i.e., prioritization of speed or accuracy), could have varied based on our instruction set (i.e., to “move as quickly and accurately as possible”; see Adam, 1992), we sought to evaluate the influence of participant strategy on violations of unitary MT/ID relations across amplitude and width manipulations in a secondary analysis.

## Secondary data reduction and analyses

Upon initial screening of our data, we noticed a wide array of error rates across individuals. An error was defined as a movement endpoint falling outside the target area and error rates ranged from 0 to 10% of the trials across participants. Although some researchers have discarded data from individuals who exhibited large error rates (e.g., Hatfield et al., 2010 excluded data from participants with error rates larger than 5%), we opted to segregate participants into two groups (i.e., accuracy-prone vs. speed-prone). Using the same 5% criterion as Hatfield et al. (2010), 12 participants exhibited error rates of 5% or below in both sessions (i.e., accuracy-prone), which included all three left-handed individuals, whereas seven participants made endpoint errors on more than 5% of the trials in either session (i.e., speed-prone). The individuals assigned to the latter group appeared to prioritize speed over accuracy, which has been previously denoted as a subjective-level of speed-accuracy trade-off performance (e.g., Adam, 1992; Zhai et al., 2004). All individuals were included in the analyses and a between-subject factor called Strategy was introduced (accuracy-prone (*n* = 12); speed-prone (*n* = 7)]. It was hypothesized that the provision of spatial terminal feedback may have only significantly influenced the performance of participants who prioritized accuracy over speed. As such, the violation of Fitts' theorem that persisted (i.e., *b*_amplitude_ slopes > *b*_width_ slopes) in the presence of spatial terminal feedback could theoretically be alleviated when assessing participants who prioritized accuracy separately from those who prioritized speed.

For statistical analyses, MT, CE and VE data were submitted to 2 Strategy (accuracy-prone and speed-prone) by 2 TF Condition (TF-Absent and TF-Present) by 4 Amplitude (155, 190, 266, and 380 mm) by 4 Width (20, 30, 40, and 50 mm) mixed-model ANOVAs. *Post-hoc* procedures for these variables were consistent with the primary set of analyses with the addition of Bonferroni corrected multiple comparisons for between groups comparisons where applicable. Data computed for each set of slopes (i.e., *b*_Fitts_ and *b*_We_) were analyzed separately via 2 Strategy (accuracy-prone and speed-prone) by 2 TF-Condition (TF-Absent and TF-Present) by 2 Component Slope (*b*_amplitude_ and *b*_width_) by 4 Average ID mixed-model ANOVAs. Where applicable, significant effects in the slopes analyses were further analyzed using Bonferroni corrected multiple comparisons. Importantly, to avoid redundancy, only significant effects involving the between-subjects factor Strategy have been reported below.

## Secondary results

### Movement time

**Figure 6:**
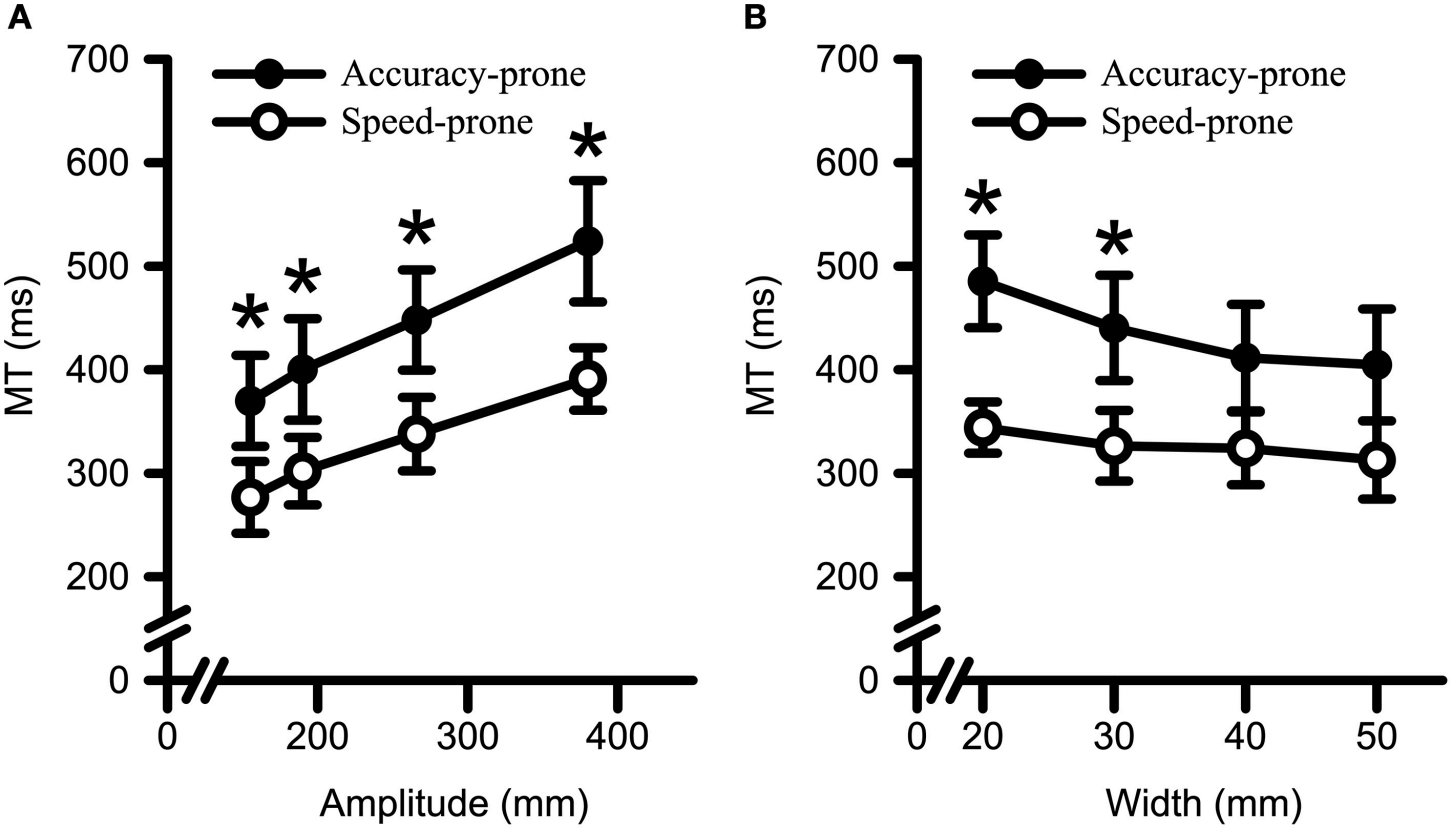
**Movement time data for both Strategy groups as a function of target (A) amplitude and (B) width**. Error bars represent ±2 SEM. Both groups exhibited longer MTs to larger target amplitudes and to smaller target widths. ^*^Denotes a significant between group difference at *p* < 0.05.

Regarding MT data (see Figure 6), a main effect was found for Strategy [*F*_(1, 17)_ = 9.67, *p* = 0.006, η^2^_G_ = 0.30]. In addition there were two significant interactions: Strategy by Amplitude [*F*_(1.5, 25.5)_ = 5.42, *p* = 0.017, η^2^_G_ = 0.01] (see Figure 6A); and Strategy by Width [*F*_(1.5, 24.5)_ = 9.24, *p* = 0.003, η^2^_G_ = 0.02; see Figure 6B]. *Post-hoc* polynomial contrast analysis of the Strategy by Amplitude interaction revealed that average MT for both groups increased with a significant linear trend with increasing target amplitude [accuracy-prone: *F*_(1, 11)_ = 179.70, *p* < 0.001, η^2^_G_ = 0.33; speed-prone: *F*_(1, 6)_ = 507.70, *p* < 0.001, η^2^_G_ = 0.52]; further, all levels of amplitude exhibited significant between-group differences (*p*s < 0.047). Decomposition of the Strategy by Width interaction with polynomial contrasts revealed that for the accuracy-prone group average MT decreased with a linear [*F*_(1, 22)_ = 81.85, *p* < 0.001, η^2^_G_ = 0.12] and a quadratic trend [*F*_(1, 22)_ = 7.91, *p* = 0.010, η^2^_G_ = 0.01] with increasing target width. However, the speed-prone group only showed a significant linear trend [*F*_(1, 6)_ = 19.96, *p* = 0.004, η^2^_G_ = 0.06]. Also, reliable between-group differences resulted from longer MT to the 20 and 30 mm target Widths for the accuracy-prone group relative to the speed-prone group (*p*s < 0.023).

### Constant and variable error

**Figure 7:**
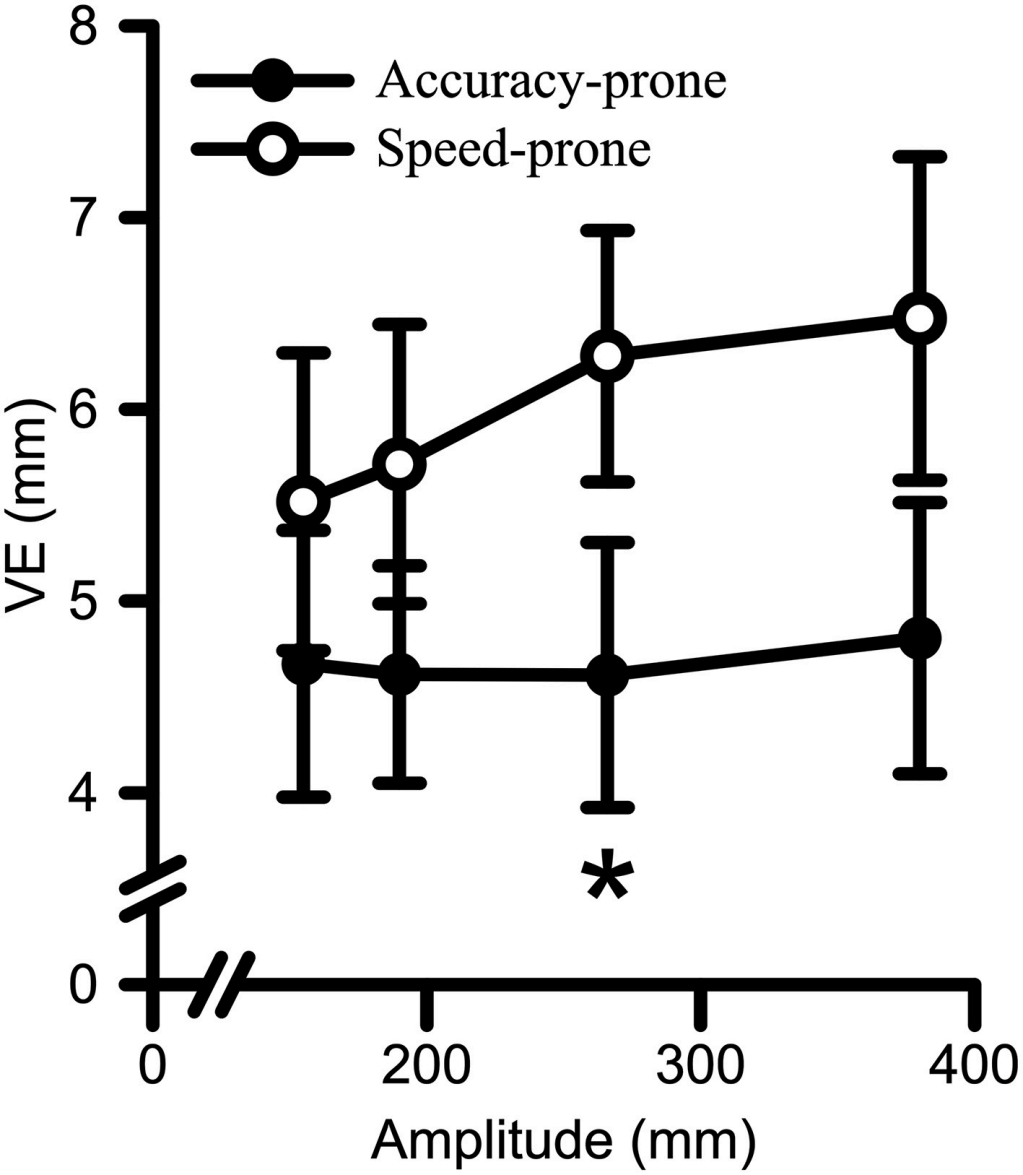
**Variable error (i.e., VE) data for both Strategy groups as a function of target amplitude**. Error bars represent ±2 SEM. Participants in the speed-prone group exhibited larger VE to larger target amplitudes. Conversely, the accuracy-prone group did not exhibit significant scaling to target amplitude. ^*^Denotes a significant between group difference at *p* < 0.05.

CE analyses revealed no main effects or interactions (*F*s < 2.73, *p*s > 0.067). The analyses of VE yielded a main effect for Strategy [*F*_(1, 17)_ = 6.07, *p* = 0.025, η^2^_G_ = 0.12], and a Strategy by Amplitude interaction [*F*_(3, 51)_ = 2.81, *p* = 0.049, η^2^_G_ = 0.01] (see Figure 7). Decomposition of the Strategy by Amplitude interaction with polynomial contrasts indicated that VE increased with a linear trend with increasing target amplitude for the speed-prone [*F*_(1, 6)_ = 21.70, *p* = 0.003, η^2^_G_ = 0.10] but not for the accuracy-prone group [*F*_(1, 11)_ = 0.65, *p* = 0.438, η^2^_G_ < 0.01]. Only one significant between group difference emerged, namely the speed-prone group exhibited a larger VE as compared to the accuracy prone group for the 266 mm Amplitude (*p* = 0.034). The same between group comparison at the 380 mm amplitude approached statistical significance (i.e., *p* = 0.054).

### Slopes analyses: *b*_Fitts_ and *b*_We_

**Figure 8:**
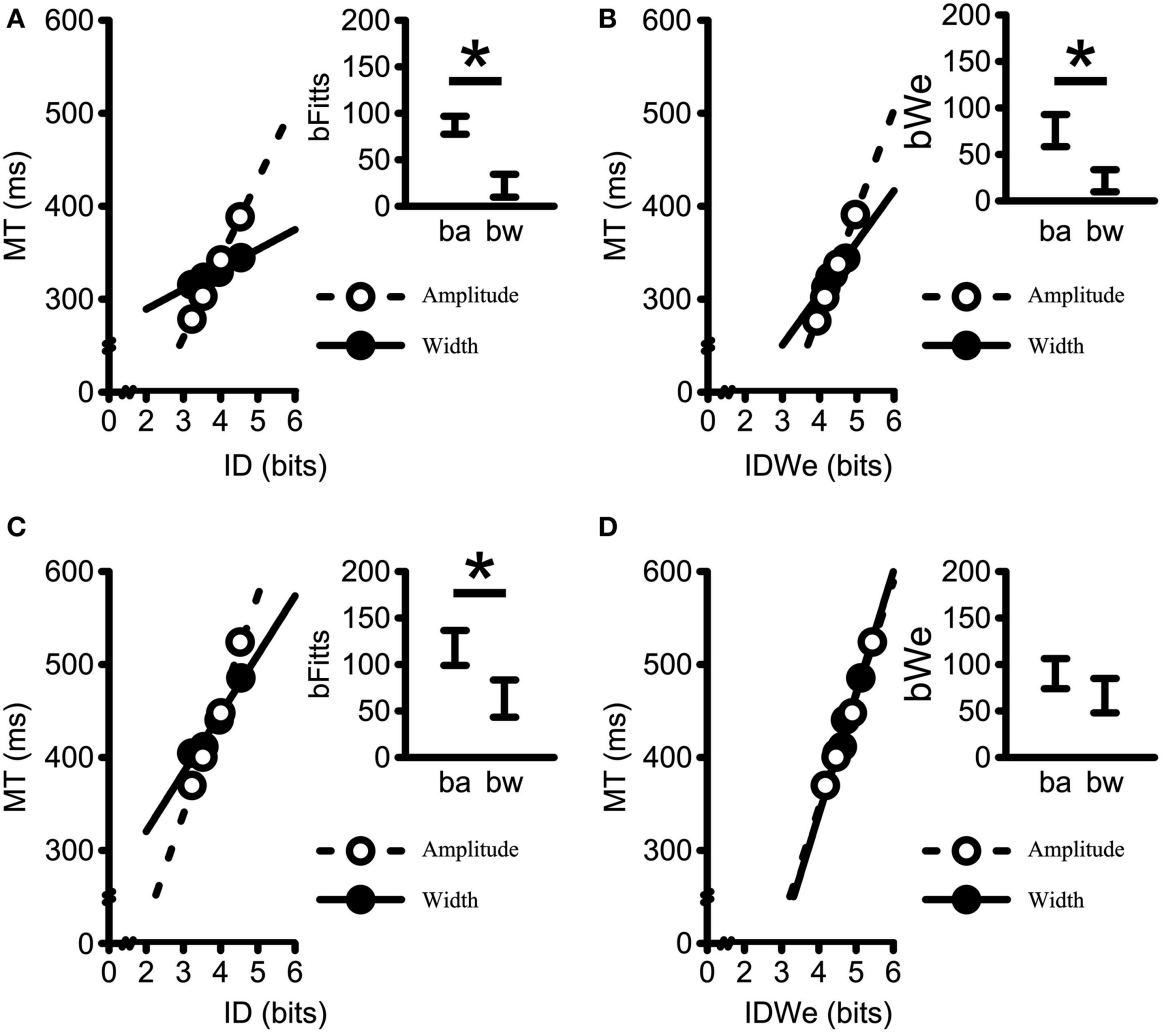
**Mean movement times plotted against (A,C) average ID (i.e.,** ***b*****_Fitts_); and (B,D) ID_We_, (i.e.,** ***b*****_We_) for both Strategy groups**. The speed-prone group is represented in **(A,B)** whereas the accuracy-prone group is represented in **(C,D)**. Data points generated by collapsing across terminal feedback, amplitudes within widths, and widths within amplitudes. Dashed lines represent linear regressions on the Amplitude data; Solid-lines represent linear regression on the Width data. All *R*^2^-values > 0.95. Insets represent the 95% confidence interval of the average within participant slopes (*b*_a_ = *b*_amplitude_; *b*_w_ = *b*_width_). The movement time scaling for the speed-prone group to changes in amplitude resulted in steeper slopes as compared to the movement time scaling to changes in width for both the standard formulation of Fitts' Law (i.e., *b*_Fitts_) and Fitts' Law when using the the effective target width formulation (i.e., *b*_We_) (i.e., **A,B**). The accuracy-prone group exhibited a comparable difference for the *b*_Fitts_ **(C)**, but not for the *b*_We_ slopes **(D)**. ^*^Denotes a significant difference at *p* < 0.05.

Figures 8A–D presents the influence of amplitude- and width-based manipulations on MT/ID relations across levels of Strategy. Figures 8A,B represent the *b*_Fitts_ and the *b*_We_ relationships for the speed-prone group. Figures 8C,D represent the same two relationships for the accuracy-prone group. As in Figure 5, the main graphs represent the mean MT vs. ID (Figures 8A,C) data and mean MT vs mean ID_We_ (Figures 8B,D) data across amplitudes and across widths. The insets again represent the 95% confidence intervals of the slopes associated with amplitude- and width-based manipulations. Analysis of the *b*_Fitts_ slopes yielded significant main effects of Strategy [*F*_(1, 17)_ = 16.26, *p* < 0.001, η^2^_G_ = 0.15], and Component slope [*F*_(1, 17)_ = 29.37, *p* < 0.001, η^2^_G_ = 0.22]. The *b*_Fitts_ relationship was steeper for the accuracy-prone group (*M* = 91 ms/bit, *SD* = 22) than for the speed-prone group (*M* = 55 ms/bit, *SD* = 10); and steeper for the amplitude-based manipulations (*M* = 106 ms/bit, *SD* = 28) as compared to width-based manipulations (*M* = 48 ms/bit, *SD* = 33). Importantly, this demonstrated that non-unitary MT/ID relations persist across participant Strategy when utilizing Fitts' theorem (i.e., *b*_Fitts_).

Conversely, the analysis of the *b*_*We*_ slopes yielded main effects of Strategy [*F*_(1, 17)_ = 9.55, *p* = 0.007, η^2^_G_ = 0.05], and Component slope [*F*_(1, 17)_ = 8.02, *p* = 0.011, η^2^_G_ = 0.02], plus a Strategy by Component Slope interaction [*F*_(1, 17)_ = 4.94, *p* = 0.040, η^2^_G_ = 0.01]. The slope values for the speed-prone group were larger for *b*_amplitude_ (76 ms/bit, *SD* = 18) than for *b*_width_(22 ms/bit, *SD* = 13) (*p* = 0.003), whereas the slope values for the accuracy-prone group did not reliably differ across *b*_amplitude_ (90 ms/bit, *SD* = 27) and *b*_width_(67 ms/bit, *SD* = 30) (*p* = 0.104). Furthermore, the *b*_width_ slopes were significantly different between Strategy groups (*p* = 0.002). Conversely, the *b*_amplitude_ slopes were not significantly different between groups (*p* = 0.733). Thus, participant Strategy and effective target width appear critical factors to consider in order to replicate unitary MT/ID relations for discrete reaching movements.

## Secondary discussion

### Strategy-based performance differences

Adam (1992) found that when instructed to move “quickly and accurately,” some individuals prioritized speed, whereas others prioritized accuracy. Accordingly, participants in the current study were subdivided into speed-prone and accuracy-prone groups, based on a 5% error cut-off. The 5% cut-off criterion was adopted because of previous use in the literature (i.e., Hatfield et al., 2010), and the notion that the generation of error rates above 4% have been argued to be in violation of the theoretic assumptions of Fitts' theorem (see MacKenzie, 1992). As such, the rationale to segregate the groups was empirically and theoretically supported. This segregation led to one analysis that did not yield steeper amplitude-based than width-based slopes.

Overall, the inclusion of the grouping factor of participant strategy resulted in differences in performance for both the MT and the VE data. Indeed, significant Strategy by Amplitude interactions for MT and VE were found in addition to a significant Strategy by Width interaction for MT. These findings revealed that participants in the two strategy groups were differentially trading-off speed and accuracy in response to changes in target amplitude and target width. Contrary to our hypothesis, however, was the finding that the provision of spatial terminal feedback did not significantly interact with Strategy for either MT or VE. Thus although the provision of feedback did reduce MTs, it did not significantly alter the scaling of speed-accuracy trade-offs.

Unitary MT/ID relations between amplitude- vs. width-based manipulations were attained from the accuracy-prone group when using the effective target width (i.e., *b*_We_). Specifically, while the speed-prone group exhibited smaller slopes for width than for amplitude manipulations for the *b*_We_ analyses, the accuracy-prone group did not exhibit different MT/ID_We_ slopes when contrasting amplitude vs. width manipulations. These participants who prioritized accuracy over speed were able to achieve a consistent level of endpoint variability (i.e., VE and W_e_) across amplitudes as evidenced by the significant Amplitude by Strategy interactions for MT and VE. That is, the speed-prone participants were unable to overcome the inherent increased endpoint variability associated with their faster movements to further amplitudes (e.g., Schmidt et al., 1979). Therefore, the difference in performance between the accuracy- and speed-prone groups was potentially based in a change in error-correcting processes during the reaching movements (i.e., online control). This could be due to the fact that some participants may have tried to fit their entire finger into the target area while others have tried to bring the center of the IRED into the target area. Such a view could be eventually tested via instructions manipulations (re.: Adam, 1992). Nevertheless, the current study highlights the potential importance of accuracy mediating processes for the replication of unitary MT/ID relations for amplitude- and width-based manipulations. Indeed, Fitts' original formulation of his theorem described the motor system underlying his equation as including visual and proprioceptive feedback loops (Fitts, 1954). Ultimately, unitary MT/ID relations can be represented by Fitts' equation, but only when taking into account individual differences regarding participant strategy.

## Conclusions

Although the presence of augmented spatial terminal did influence MT, it did not significantly alter the relative influences of amplitude-based and width-based manipulations on MT scaling. Fitts' theorem was only replicated when the MT/ID slopes were calculated using effective target width, with the data from individuals prioritizing endpoint accuracy. All other combinations of conditions resulted in the replication of the violation of non-unitary slopes for amplitude- vs. width-based manipulations (i.e., greater *b*_amplitude_ compared to *b*_width_). Ultimately this study reaffirms the importance of performance strategy (i.e., Adam, 1992), and effective target width (i.e., *b*_We_; Welford, 1960) when assessing, and replicating Fitts' theorem (cf. Fitts' Law). More importantly, this study provides a novel finding that it is only when both strategy and effective target width are taken into consideration, that unitary slopes for amplitude- vs. width-based manipulations can be observed. It thus seems that further studies and refinements of Fitts' theorem are warranted.

### Conflict of interest statement

The authors declare that the research was conducted in the absence of any commercial or financial relationships that could be construed as a potential conflict of interest.

## Footnotes

^1^Although many might correctly argue that the possessive of Fitts should spell “Fitts's,” we employ Fitts' to facilitate pronunciation and readability.

^2^Although the IDs did differ across and within the *b*_amplitudes_ and the *b*_width_, ID averages were employed to generate the Average ID factor levels for the ANOVA (see Table 1). Also, the actual average ID levels were retained for the *b*_We_ analyses to provide meaningful contrasts between ANOVAs.
